# Prevalence of Chronic Kidney Disease in Adults with Type 2 Diabetes Mellitus from Oxcutzcab, Yucatán

**DOI:** 10.5195/cajgh.2019.379

**Published:** 2019-05-10

**Authors:** Andrea Muñoz Lara, Jorge García Vega, Juan Pablo Moncada Patiño, Alma Rosa Tovar, Patricia Isolina del Socorro Gómez Aguilar

**Affiliations:** 1 University of Guanajuato, Mexico; 2 Autonomous University of Yucatán, Mexico

**Keywords:** Glomerular filtration rate, Chronic kidney disease, Type 2 diabetes mellitus, Glomerular filtration rate

## Abstract

**Introduction:**

The complications of type 2 diabetes mellitus (T2DM), such as chronic kidney disease (CKD), are the second leading cause of death in Oxcutzcab municipality of Yacatan, Mexico. The objective of the study was to estimate the burden of chronic kidney disease in a sample of patients with T2DM from Oxcutzcab municipality of Yacatan, Mexico, region characterized by high amound of poverty and vulnerabidity.

**Methods:**

This is a descriptive study involving 108 adult patients between 26 and 79 years old with T2DM who attended the PROSPERA, social protection program under the direction of Ministry of Social Development of Mexico (88% female and 12% male). Weight, height, BMI, and years of post T2DM diagnosis were measured. Estimated glomerular filtration rate (eGFR) was calculated using the Cockcroft-Gault formula.

**Results:**

We found that 39.81% of participants had stage one kidney damage, 34.26% stage two, 24.07% stage three, one case of stage four, and one of stage five. BMI measurements indicated that 40.74% of participants were obese (≥30kg/m^2^), 35.19% were overweight, and 1.85% were underweight. In terms of years since diagnosis, 37.04% of the participants were diagnosed five years ago and less, 29.63% of participants were diagnosed 6-10 years ago, 22.22% between 11-15 years ago, 8.33% between 16-20 years ago, and 2.78% of participants over 20 years ago.

**Conclusions:**

Most participants were in stages one to three of kidney damage, where the main objective of the medical team was medical treatment of T2DM and comorbidities, as well as nutritional support to prevent further complications. There was only one case in stage four and five each, where dialysis and kidney transplantation became necessary. Both cases presented had a history of T2DM for over 20 years. It is important to identify early kidney damage to improve quality of life, reduce the treatment costs, and lower mortality.

